# Effects of the Preschool-Based Family-Involving DAGIS Intervention Program on Children’s Energy Balance-Related Behaviors and Self-Regulation Skills: A Clustered Randomized Controlled Trial

**DOI:** 10.3390/nu12092599

**Published:** 2020-08-26

**Authors:** Carola Ray, Rejane Figuereido, Henna Vepsäläinen, Reetta Lehto, Riikka Pajulahti, Essi Skaffari, Taina Sainio, Pauliina Hiltunen, Elviira Lehto, Liisa Korkalo, Katri Sääksjärvi, Nina Sajaniemi, Maijaliisa Erkkola, Eva Roos

**Affiliations:** 1 Folkhälsan Research Center, Topeliuksenkatu 20, FI-00250 Helsinki, Finland; rejane.fig@gmail.com (R.F.); reetta.lehto@folkhalsan.fi (R.L.); riikka.pajulahti@helsinki.fi (R.P.); essi.skaffari@helsinki.fi (E.S.); taina.sainio@helsinki.fi (T.S.); pauliina.hiltunen@folkhalsan.fi (P.H.); elviira.lehto@helsinki.fi (E.L.); eva.roos@folkhalsan.fi (E.R.); 2 Department of Food and Nutrition, University of Helsinki, P.O. Box 66, FI-00014 Helsinki, Finland; henna.vepsalainen@helsinki.fi (H.V.); liisa.korkalo@helsinki.fi (L.K.); maijaliisa.erkkola@helsinki.fi (M.E.); 3 Clinicum, Faculty of Medicine, University of Helsinki, FI-00014 Helsinki, Finland; 4 Department of Teacher Education, University of Helsinki, P.O. Box 9, FI-00100 Helsinki, Finland; katri.saaksjarvi@helsinki.fi (K.S.); nina.sajaniemi@helsinki.fi (N.S.); 5 School of Applied Educational Science and Teacher Education, Philosophical Faculty, University of Eastern Finland, P.O. Box 111, FI-80101 Joensuu, Finland; 6 Department of Public Health, Clinicum, P.O. Box 63, University of Helsinki, FI-00014 Helsinki, Finland

**Keywords:** energy balance-related behaviors, self-regulation skills, preschoolers, children, randomized controlled trial, intervention effects, parental educational level, intervention mapping, multicomponent intervention

## Abstract

The study examines the effects of a preschool-based family-involving multicomponent intervention on children’s energy balance-related behaviors (EBRBs) such as food consumption, screen time and physical activity (PA), and self-regulation (SR) skills, and whether the intervention effects differed among children with low or high parental educational level (PEL) backgrounds. The Increased Health and Wellbeing in Preschools (DAGIS) intervention was conducted as a clustered randomized controlled trial, clustered at preschool level, over five months in 2017–2018. Altogether, 802 children aged 3–6 years in age participated. Parents reported children’s consumption of sugary everyday foods and beverages, sugary treats, fruits, and vegetables by a food frequency questionnaire, and screen time by a 7-day diary. Physical activity was assessed by a hip-worn accelerometer. Cognitive and emotional SR was reported in a questionnaire by parents. General linear mixed models with and without repeated measures were used as statistical methods. At follow-up, no differences were detected in EBRBs or SR skills between the intervention and control group, nor did differences emerge in children’s EBRBs between the intervention and the control groups when stratified by PEL. The improvement in cognitive SR skills among low PEL intervention children differed from low PEL control children, the significance being borderline. The DAGIS multicomponent intervention did not significantly affect children’s EBRBs or SR. Further sub-analyses and a comprehensive process evaluation may shed light on the non-significant findings.

## 1. Introduction

Young children’s food intake, screen time, and physical activity (PA), commonly referred to as energy balance-related behaviors (EBRBs) [1], are of importance since they can predict the future weight status and health of children [2,3,4]. A socio-economic status (SES) gradient exists already in preschoolers’ EBRBs; those with low SES family backgrounds tend to have less healthy EBRBs such as higher intake of sugary foods or beverages and excessive screen time [5,6,7].

Home and an early childhood education and care center, hereafter preschool, are the settings where three to six-year-olds spend most of their time, and it is therefore important that these environments promote healthy EBRBs including sufficient PA and fruit and vegetable (FV) consumption [8,9,10]. Reviews have concluded that EBRB interventions should be conducted at preschools and homes simultaneously in order to be successful [11,12]. Preschool-based family-involving interventions have been reported to be promising [12,13,14,15], although some studies show no effects on EBRBs [12,14,16]. This has raised discussion on intervention design and implementation in families [12]. When designing interventions for the general population, they should reach and show higher effects on those needing it most, namely those with low SES backgrounds [5,17]. To date, knowledge of the equity effectiveness of EBRB interventions among children is sparse [18,19]. Promoting several EBRBs simultaneously is challenging, as the aim can be to both promote healthy behaviors and discourage unhealthy behaviors. Strategies can differ, a review concluding that promoting PA among young children is successful when focusing on the preferred behavior, rather than focusing on decreasing sedentary time such as lying or sitting down [20].

Strengthening children’s self-regulation (SR) skills in parallel to promoting children’s healthy EBRBs could be an effective strategy in interventions [21,22]. Self-regulation is a multidimensional concept, briefly described as the capacity of a goal-directed behavior to regulate actions, emotions, and cognitions [23]. Cognitive SR skills refer to executive functioning such as self-monitoring to plan and proceed toward long-term goals [24,25,26], whereas emotional SR skills refers to capacities such as being able to recognize one’s own feelings and staying calm in stressful situations [24,25]. Associations between children’s SR skills and less favorable EBRBs and weight status have been found [21,22,24,25]. The Head Start study tested the strategy of strengthening young children’s SR skills alongside promoting their healthy EBRBs [27]. The intervention included four arms: intervening on EBRBs and SR skills; intervening on EBRBs; intervening on SRs skills; and no intervention. Effects were seen in lower sugar-sweetened beverage consumption in the study arm promoting EBRBs and SR skills compared with the other arms [27].

The Increased Health and Wellbeing in Preschools (DAGIS) intervention aimed to promote preschoolers’ (aged 3–6 years) healthy EBRBs and SR skills. The assumption was that there would be greater effects on children from families with low parental educational levels (PEL), also assuming a reduction in any health gaps between children with low and high PEL backgrounds [28]. The intervention development process was guided by the Intervention Mapping (IM) framework [29] and the process is described elsewhere [28]. A cross-sectional study served as the needs assessment [7,28], and based on these findings, there were three main aims: to reduce children’s screen time; to reduce the consumption of sugary everyday foods and beverages; and to increase vegetable consumption. In these three behaviors, the needs assessment showed less favorable behaviors among children with low PEL background [28]. To promote alternatives to the reductions, additional aims were to increase fruit and berry consumption and total PA (light, moderate, and vigorous intensity) [28]. In addition, the intervention aimed to strengthen children’s SR skills. Activities were planned to suit families with low PEL backgrounds.

In Finland, 78–86% of three to six year-olds attend municipality-driven preschools [30]. Therefore, preschools offer a good setting for interventions. As screen time and sugary food and beverage consumption occurs mostly at home [31], homes were considered as an equally important intervention setting. The developed program lasted 23 weeks, and was divided into five themes: SR skills; PA; fruit and vegetables; screen time; and sugary foods and beverages. Each theme was in focus for four to five weeks.

In this study, we aimed: (1) to evaluate the effects of a preschool-based family intervention on children’s EBRBs and SR skills, and (2) to evaluate whether effects were stronger among children with low PEL background than among those with high PEL background.

## 2. Materials and Methods

The DAGIS intervention study is a preschool-level clustered randomized controlled trial (RCT) aimed to promote preschoolers’ healthy EBRBs and SR skills so that those from low SES background would benefit most from the program. The study was conducted between September 2017 and May 2018 including baseline and follow-up measurements [28]. Early educators delivered the program and all included activities to all preschoolers independently of their participation in the study. Prospective trial registration number: ISRCTN57165350 (the 8th of January 2015).

### 2.1. Recruitment

We aimed to invite municipalities that had a high number of preschools and had a large variety in educational and income levels among inhabitants as well as being located within a convenient distance from the Helsinki region. Municipalities invited were selected by comparing municipality statistics from southern and western Finland [32], and excluded municipalities that were already part of the previous comprehensive DAGIS survey in 2015–2016 [7]. Power calculations prior to the recruitment for the intervention were based on the DAGIS survey results; specifically, we used the average (about 1.7 times/week for all and about 2 times/week for low PEL group) and standard deviations of children’s sugary food and beverage consumption frequency [7]. Based on those values, we decided to aim at a decrease of 0.74 times/day in sugary foods and beverages consumption frequency. To detect a change of 0.74 times/day less sugary foods and beverages, the required sample size was calculated to be 432 children, considering an attrition rate of 70% (Fpower macro, SAS version 9.4.). The significance level was set at 5% and the power at 80%.

**Figure 1:**
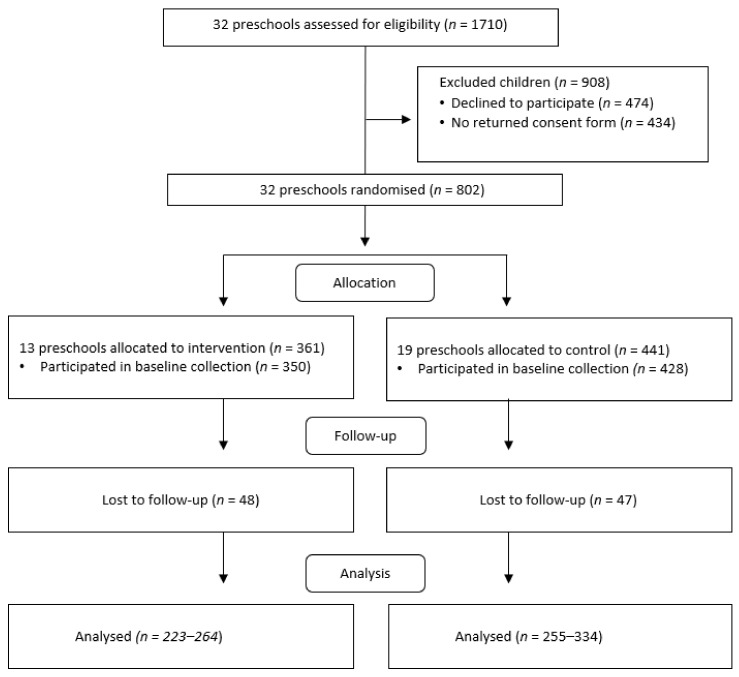
Flow chart in the Increased Health and Wellbeing in Preschools (DAGIS) intervention study, in accordance with the Consolidated Standards of Reporting Trials (CONSORT) 2010 statement [33].

Altogether, seven municipalities were invited to participate in the study, and an oral presentation on the study was offered. Five municipalities had an oral presentation; two of these municipalities chose to participate. One municipality decided that all of its preschools (*n* = 29, preschool managers *n* = 19) would participate, whereas the other municipality allowed its preschool managers to make the decision individually, as such, the managers of three preschools chose to participate. We decided that these 32 preschools and 1702 eligible preschoolers were sufficient for our study (Figure 1).

Researchers visited each preschool to inform early educator professionals about the project and their role in the project. The recruitment phase lasted 1–2 weeks, and families returned informed consents (or refusals to participate) to preschools in sealed envelopes. Thereafter, the researchers returned to preschools to distribute the baseline research material for early educators, parents, and children.

### 2.2. Ethical Issues

The DAGIS intervention study received ethics approval from the Helsinki Ethics Review Board in humanities and social and behavioral sciences (22/2017; 16 May 2017). Early education professionals were informed about the study through site visits. The early educators’ questionnaire stated that participation was voluntary and that the early educators had the option to withdraw at any stage of the study. Early educators gave their consent by filling in the questionnaire. Families returned written informed consent, and thereafter, the questionnaires were delivered.

### 2.3. Data Collection and Measurements

The baseline data collection occurred in four waves over five weeks and the follow-up data collection in three waves over five weeks. Data collection in waves was necessary due to the limited number of accelerometers available for measuring children’s PA. Research staff visited each preschool to instruct early educators and left printed screen time diaries for families, study questionnaires for families who had requested paper copies, and accelerometers for children. These materials were picked up from preschools one week later. However, most parents requested that their questionnaires be sent electronically by sending the parent’s main questionnaire as a personal link and the food frequency questionnaire link by email.

#### 2.3.1. Measurements

Screen time was assessed by a printed screen time diary. In the diary, parents recorded their child’s use of screens outside preschool time whenever the child used a screen for more than 10 min in a row. Screen use was recorded separately for different screens: TV, DVD, computer, tablet, or cell phone. The screen time diary was a slightly modified version from a previous validated diary [34], as the original did not include portable screens and questions about screen contexts. The screen time diary has shown good reproducibility [35]. Screen time was calculated for children who presented data for at least three weekdays, and one weekend day. Total screen time (min/day) was calculated as a weighted mean: (5 × weekday mean + 2 × weekend mean)/7.

Children’s PA was assessed by a hip-worn accelerometer, the ActiGraph wGT3X-BT (ActiGraph, LLC, Pensacola, FL, USA), 24 h/day over seven consecutive days, and parents kept a screen time diary over the same days. A 15-s epoch length was used for data derived from accelerometers, and more than ten minutes of consecutive zeroes was set as non-wearing time [36]. In the analyses, the cut-off points of Evenson et al. [37] for children aged 5–15 years were used, which means that total PA including light, moderate, and vigorous intensity PA is defined as more than 100 counts/min. Inclusion criteria for the child’s PA data to be in the analyses were that there were data for at least four days, of which one was a weekend day. In addition, each day needed to have 600 min or more of awake wearing time. The mean total PA (min/day) was used in the analyses.

The original 47-item food frequency questionnaire (FFQ) was designed for the DAGIS survey to particularly measure the consumption frequencies of vegetables and fruits as well as sugary foods and beverages [38]. It has shown acceptable validity for ranking food group consumption compared with 3-day food records [38], and testing the reproducibility of the items has yielded acceptable results [35]. In the DAGIS intervention, the FFQ was expanded into a 51-item FFQ that included six food groups (vegetables, fruit, and berries; dairy products; fish meat and eggs; cereal products; beverages; and other foods such as sweets and snacks). A link to the electronic 51-item FFQ was sent to all parents and hard copies were sent to those who did not fill in the electronic version. Parents reported how many times during the past week the child had consumed foods outside preschool hours. The FFQ included three answer options: not at all, times per week, and times per day. The instruction was to either tick the ‘not at all’ box or to write a number in one of the other columns. The FFQ was intentionally restricted to not cover municipality-provided foods and beverages consumed during preschool hours because parents would not have been able to reliably report these foods.

The three food consumption frequency variables (‘sugary everyday foods and beverages’, ‘sugary treats’, and ‘fruit and vegetables (FV)’) were formed by summing up the consumption frequencies (times/week). The sugary everyday foods and beverages variable included flavored yogurt and quark; puddings; sugar-sweetened cereals and muesli; berry, fruit, and chocolate porridge with added sugar; berry and fruit soups with added sugar; soft drinks; flavored and sweetened milk- and plant-based beverages; and sugar-sweetened juices. The sugary treats variable included ice cream, chocolate, sweets, cakes, cupcakes, sweet rolls, Danish pastries, pies and other sweet pastries, and sweet biscuits and cereal bars. The FV variable included fresh vegetables, cooked and canned vegetables, fresh fruit, and fresh and frozen berries.

Children’s SR skills were assessed with 10 items derived from the Child Social Behavior Questionnaire, previously used in the Millennium Cohort Study on 3-year-olds [26]. Five items assessed cognitive skills and five items emotional SR skills. Each statement had three response options: disagree; agree to some extent; and fully agree. The mean points for each sub-dimension were calculated and used in the analyses. The internal consistency reliability as Cronbach’s alphas was 0.68 for cognitive and 0.78 for emotional SR skills.

#### 2.3.2. Parental Educational Level

The parent filling in the guardian’s questionnaire reported his/her own highest educational achievement and the education of a partner living in the same household. The six answer options were categorized as follows: low educational level (comprising comprehensive school, vocational school, or high school); middle educational level (bachelor’s degree or college); and high educational level (master’s degree or licentiate/doctor). The highest educational level among parents was used as the parental educational level (PEL) variable in the analyses. In four cases, the highest education was not the education level of the mother or the father of the child, but that of a spouse living in the same household.

#### 2.3.3. Confounding Factors

The parent reported the date of birth and gender of the participating child. In the statistical analysis, adjustments were made for the child’s gender and age at baseline (continuous) for the categorical variable PEL and for the municipality.

### 2.4. Randomization, the Intervention, and the Program Content

Randomization was made at the preschool manager-level, separately for the two municipalities by an online randomization program (https://www.randomlists.com/team-generator). Preschools were divided into small and large preschools before randomization. After the baseline measurements, preschools were informed whether they had been randomized into the intervention (*n* = 13) or control (*n* = 19) group (Figure 1).

In intervention preschools, all early educators received program training. The training was split into a longer training session after the baseline measurements and a shorter training session around the middle of the 23-week program, in all, approximately 8 h [28]. Throughout the intervention, two researchers engaged with early educators conducting the program by email. Basically, the program at preschools was based on the international MindUp™ program [39]. Healthy EBRBs promoting strategies and methods were added to the existing ones in the program, and a program for families was developed [28]. The program was run in both preschools and homes and divided into five themes, all of which lasted 4–5 weeks: SR skills; physical activity; fruit and vegetables; screen time; and sugary foods and beverages. SR skills along with each EBRB were emphasized throughout the program in the preschool activities. SR skills were promoted by brain breaks, which were a few minutes’ calming down and breathing sessions three times per day, led by early educators. In addition, early educators were trained to teach children to recognize and reflect on different feelings. In the family activities, focus was set on the children’s EBRBs, and on how parents, by acting as role models and changing the availability and accessibility of the home environment, could influence their children’s EBRBs. The methods used for families were, among others, information letters, emails containing videos or articles, bingos related to EBRBs, and two fairy tales written for the project. For each of the five themes, preschools arranged one activity afternoon. Early educators received the instructions and needed materials for the activities at the program training sessions. The activity afternoons were conducted as a workshop for children and parents to which all families were invited. An activity afternoon could consist of a working sheet about vegetable eating habits and favorite vegetables, or a vegetable tasting session that children and parents conducted together. Materials that were produced during the afternoons were expected to be displayed at the preschool, so that families could see each other’s works. The early educators in the control preschools received training for the program after the intervention was finished.

### 2.5. Statistical Analyses

Differences between the participants’ characteristics and the two groups (intervention/control) at baseline were analyzed by the Chi-square test (categorized variables) and *t*-test (continuous variables). Our main outcomes were total screen time (min/day), total PA (min/day), two variables related to sugar consumption (sugary everyday foods and beverages, and sugary treats, as times/week), total FV consumption frequency (times/week), and SR skills (cognitive and emotional dimensions, as scores). As a first step, a simple model was used to show the comparison between the intervention and control groups. To evaluate this, we used the general linear mixed models adjusted for baseline value of the outcome. This first model was used as a simple description of the results at follow-up. As a second step, a more complete and appropriate model was used with the major interest to evaluate the results between follow-up and baseline for the control and intervention groups. For this aim we used the linear mixed models with repeated measures for all outcomes, taking into account the interaction between the two groups and two time-points of baseline and follow-up. In the mixed models, normal distribution was visually checked. The preschool unit was used as a random effect in order to adjust for variability between the preschools. All aforementioned analyses were adjusted for child’s gender, age at baseline, municipality, and PEL. Furthermore, accelerometer wearing time was included as an adjustment variable in the analyses where PA was the outcome. We also evaluated linear mixed models with three-level interactions: groups (intervention and control), time-points (baseline and post-intervention), and PEL. For these models, the results for the comparison between the two groups and time-points were presented as stratified by PEL group. In all analyses, multiple imputation was applied for independent variables with missing values. The number of children included in the analysis of each dependent variable and the missing values are presented in Supplementary Table S1 and the complete results for the linear mixed models with repeated measures and the respective effect size for interaction is presented in Supplementary Table S3.

All analyses were based on the intention-to-treat principle so that all randomized participants were included in the analysis in their randomized intervention group. General statistical analysis was performed and tables created using SPSS version 25. Mixed models, effect size for models’ interaction, and multiple imputation analysis were conducted in R version 3.4.3 using the lme4, MuMIn, and MICE packages, respectively. For all analyses, a 5% statistical significance level was adopted.

## 3. Results

The average age of children in the study was 5.24 (±1.06) and 5.14 (±1.04) years for the control and intervention groups, respectively. Even though most characteristics were similar in the groups, a higher percentage of children with high educational level parents were found in the control group (26%) than in the intervention group (18%) (Table 1).

Table 2 shows the descriptive results for children’s EBRBs and SR skills according to the intervention and control group, at baseline and at follow-up, whereas the corresponding results according to PEL are presented in Supplementary Table S2. Children had about the same daily screen time in the intervention and control groups at baseline (Table 2), but low PEL children had higher screen time than the other groups (Supplementary Table S2). The FV consumption at baseline was higher in the high PEL groups than in the other groups (Supplementary Table S2).

**Figure 2:**
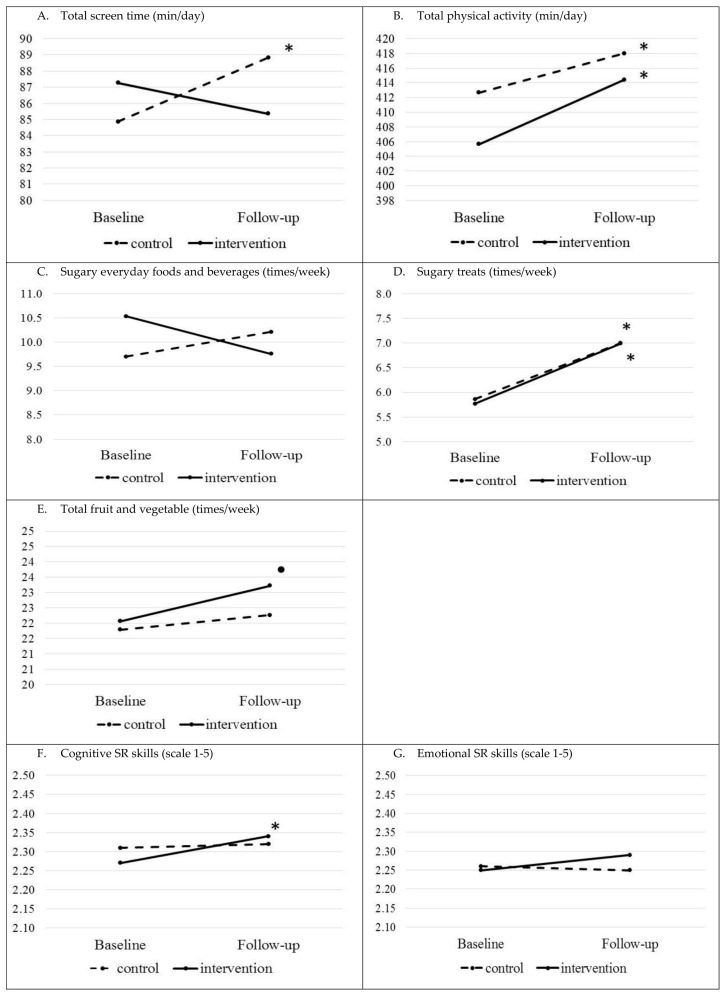
Children’s EBRBs (heading (**A**–**E**)) and SR skills (headings (**F**,**G**)) at the baseline and follow-up in the intervention and control groups (means). For exact mean values, please see Table 2 (* *p*-value < 0.05, • *p*-value < 0.01 for the difference between the follow-up and baseline within the group).

**Figure 3:**
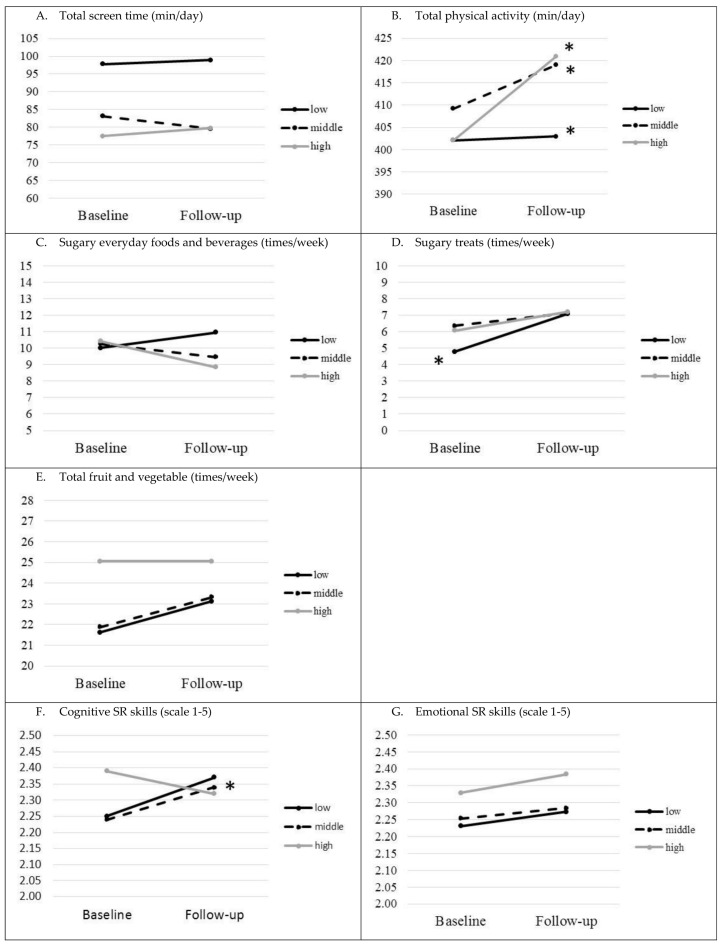
Children’s EBRBs (headings (**A**–**E**)) and SR skills (headings (**F**,**G**)) within the intervention group separated by highest parental educational level (PEL) (means). For exact mean values, please see Supplementary Table S2 (* *p*-value < 0.05 for difference between follow-up and baseline within the group).

Table 3 shows the comparison between the intervention and control groups at follow-up adjusted for respective baseline outcome values. Figure 2 and Figure 3 present the mean of the main outcomes (descriptive values from Table 2) at the baseline and follow-up for the intervention and control groups, and for the PEL subgroups of the intervention group.

There were no significant differences detected in follow-up between the intervention and control groups for children’s total screen time, total PA, consumption frequencies of sugary everyday foods and beverages, sugary treats, and FV, and cognitive and emotional SR skills (Table 3).

The results between the baseline and follow-up within the control and intervention groups differed for some EBRBs and SR skills (Table 3, see means in Figure 2). In the intervention group, the change between baseline and follow-up in total screen time was not significant, whereas there was a significant increase, approximately 4.5 min/day, in screen time in the control group (*p* = 0.028, Table 3, Figure 2A). The control group significantly increased in total PA on average by 24 min/day (*p* < 0.001), and the intervention group had a significant increase of 27 min/day (*p* < 0.001, Table 3 and Figure 2B). There was an increase in sugary treat consumption frequency in both groups (*p* < 0.001 in both groups, Table 3). In the intervention group, there was a trend, albeit not significant (*p* = 0.088), where FV consumption frequency increased (Table 3, Figure 2E). A positive significant change in points in cognitive SR skills was observed in the intervention group (*p* = 0.011, Table 3, Figure 2F).

Similar comparisons of children’s EBRBs and SRs skills at follow-up stratified by PEL and the comparison between baseline and follow-up for intervention and control groups stratified by PEL are presented in Table 4. To illustrate the results within the separate PEL intervention groups, figures are presented with the mean of main outcomes at baseline and follow-up (Figure 3).

No significant differences were found when examining EBRBs and SR skills stratified by PEL (Table 4). In follow-up, there was a borderline significant result in cognitive SR skills when comparing low PEL intervention and control groups (*p* = 0.051).

Within the groups, the low PEL control group decreased their cognitive SR skills (borderline significance, *p* = 0.052). The total PA increased significantly within all intervention and control groups when stratified by PEL (*p* < 0.001 for all subgroups, Table 4, Figure 3B). The sugary treat consumption frequency increased within low PEL control and intervention groups (*p* < 0.001 in both groups), and in the middle PEL control group (*p* = 0.027, Table 4, Figure 3D). Cognitive SR skills strengthened in the middle PEL intervention group (*p* = 0.038, Table 4, Figure 3F).

## 4. Discussion

We detected no differences in EBRBs or SR skills between the intervention and the control group in our preschool-based family-involving RCT. Furthermore, changes in children’s EBRBs according to PEL did not differ between the intervention and control groups at follow-up, although a borderline significant result emerged in low PEL children in the intervention group, improving their cognitive SR skills compared with the corresponding control group (*p* = 0.051).

A possible reason for not detecting significant intervention effects might be that the goals set were unrealistic (0.74 times/day decrease in sugary foods and beverages), or it would have required a higher number of children. Our study was a complex multicomponent intervention of relatively short duration. Each of the five program themes were focused on for 4–5 weeks, which could have been too short a duration for changes to occur. Therefore, further evaluation of the effects is needed. Furthermore, the analysis did not show stronger intervention effects in low PEL children. Still, cognitive SR skills strengthened in the low PEL intervention group compared with the low PEL control group, and the results bordered on statistical significance. Within the low PEL control group, cognitive SR skills decreased; also here the results did border to reach statistical significance. However, a significant improvement in cognitive SR skills occurred among middle PEL intervention children. Since the above-mentioned increases in cognitive SR points when comparing control and intervention group were small, these results might lack practical implication. The Head Start intervention showed improvements in SR skills and a decrease in sugar-sweetened drink consumption in the group that received the intervention promoting both EBRBs and SR skills, compared with the other three groups [27]. Although the aims of that study and ours were similar, the results are not totally comparable. The age group in Head Start was slightly older (4–9 years), and SR skills were measured by another instrument. In both studies, activities to strengthen SR skills were mainly conducted in preschools, whereas parents were the main target when promoting healthy EBRBs. It was discussed that parents might not have been sufficiently engaged, which may have led to null results regarding the children’s EBRBs, which may also be the case in the DAGIS.

Within the intervention and control group, several significant changes occurred in the EBRBs. The control group increased their screen time by approximately 4.5 min/day, whereas no changes were detected within the intervention group. For the control group, it had about a 30 min/week higher screen time, which might eventually harm energy balance, weight status, and development of SR skills. The results of the control children followed the trend that screen time increases with age among young children [40]. The ToyBox study also did not reveal an overall positive effect on screen time [16], nevertheless when including a process evaluation, a reduction in computer/video games time was shown [14]. Subgroup analyses in ToyBox showed less TV time during weekends in the intervention girls [16], and subgroup analyses should also be considered in the DAGIS study.

The total PA increased in the control and intervention group. A recently published European study reported that moderate-to-vigorous PA increased from the age group of 2–3 years to 4–5 years, and further to 6–7 years [41]. The trend might explain the results in the DAGIS. Moreover, the follow-up occurred in spring, when there are more daylight hours than at the baseline in autumn. Studies have revealed that the higher the temperature and the more daylight present, the higher the level of PA among children [42,43]. The municipality, in which all preschools participated, simultaneously runs a training program for all early educators aimed at increasing preschool PA, which has increased all children’s preschool PA independently of intervention status. Previous interventions have reported no effects on children’s PA [44,45,46], and discussion has ensued on whether short durations such as six weeks of promoting PA are sufficient to detect an increase in children’s PA [16,47].

The follow-up results for sugary everyday food and beverage consumption outside preschool hours did not differ between the intervention and control groups. The reduction was mainly supposed to happen at home, as these foods are seldom served at Finnish preschools [31]. The program implementation in families might have been weak, leading to no changes. This needs to be further studied by analyzing the processes in the intervention. We found an increase in sugary treat consumption in both the control and intervention low PEL groups (Supplementary Table S2), but no changes in the middle or high intervention groups. It seems that as children grow older, the consumption increases, especially in low PEL groups, which might lead to a greater gap between the PEL groups. The change in FV consumption did not differ between the intervention and control groups. However, while the control group had a stable consumption of FV at both time-points, the consumption frequency in the intervention group increased by 1.3 times/week. Similarly, some intervention studies have shown improvements in FV consumption [48], although a systematic review concluded that multicomponent FV interventions have provided low evidence of increasing FV consumption [49].

When developing the DAGIS intervention, the focus was set on understanding the low educational level context and how to, by means of a universal intervention, reach those with low PEL backgrounds [28]. One strategy was to produce easy-to-read materials as the ToyBox intervention study discussed that the lack of significant results for children’s food consumption might have been due to the intervention materials being insufficiently tailored to those with low education levels [13]. The DAGIS logic model of change included primary outcomes, which were seen as the most important determinants for explaining socio-economic differences in children’s EBRBs. The main primary outcomes (i.e., adults role modeling and changes in the environment in availability and accessibility of, for example, foods and screens), should be examined next. It is more likely to see changes in these due to the relatively short duration of the intervention. Generally, it has been concluded that availability and accessibility (foods, screens) in the home environment would be of great importance for children’s health behaviors in low PEL families [13].

As this study includes the intention-to-treat effect analysis, it was assumed that all intervention preschools and families conducted the program in the same manner and at the same intensity. Further analysis including fidelity and implementation degree of the program will yield a deeper understanding of the effects. The importance of the implementation degree has been discussed in conjunction with null results in multicomponent interventions [50].

The DAGIS intervention study had limitations that should be acknowledged. The short intervention time, in all, five months, was a limitation, but the project as a whole needed to be conducted during a preschool year. Previous discussion has questioned whether a short time period is adequate for children to change their EBRBs [13,44]. In addition, children’s baseline consumption of FV, mean three times/day outside preschool time, was fairly high, which sets challenges for achieving an increase. Furthermore, reliably measuring food consumption is challenging. However, reproducibility and validity of our parental FFQ have been tested [36,38]. Still, the FFQ reflects the foods eaten during the last week outside preschool time and does not allow for analysis of whether food consumption changed at preschool. The 10-item questionnaire assessing two dimensions of children’s SR skills had three answer categories, which might not have been sensitive enough to capture changes. Many instruments are available to assess children’s SR skills, but no consensus exists on their validity in evaluating this multidimensional concept [51]. Finally, the sample size might not have been sufficiently large to detect significant results. The power calculations were conducted based on means and standard deviations from the DAGIS cross-sectional survey [7]. Some dissimilarities exist between these two studies such as the number of preschools and municipalities and the proportion of low PEL families participating, which might have led to an underpowered study.

A strength of the study is that the study development was guided by the IM framework [28], which enabled systematic planning. The logic model of change was formed on the best existing knowledge, and on a comprehensive evaluation of the Finnish preschool-family context [10,28]. This enables further systematic evaluations of the processes. The fairly high response rate of families, 47%, and having all preschools from one municipality participating including diverse preschools as well as diverse families can be seen as a strength. The high response rate indicates a lower selection bias among the participants. In addition, slightly more than 30% of the participating families had low education levels. It is often seen as a challenge that the less educated tend not to participate in intervention studies [52]. The study also included a combination of instruments such as the accelerometer for assessing PA, a validated screen time diary, and a validated FFQ for robust assessment [35,38].

The fairly new approach of simultaneously strengthening children’s SR skills and promoting their EBRBs can be seen as a strength and also as a risk. To the best of our knowledge, this approach has been evaluated in one other study [27], where it was discussed that the next step should be integrating SR skill promotion into the EBRB context. In the DAGIS study, this can be seen as a strength as the program enhanced SR skills, while simultaneously promoting EBRBs by adding more materials to the existing program. The materials and methods for the program also underwent pretesting [28].

## 5. Conclusions

The DAGIS intervention study aimed to promote preschoolers’ EBRBs and SR skills through a preschool-based family-involving intervention conducted as a clustered RCT. We detected no significant differences in the preschoolers’ EBRBs between the intervention and control groups at follow up. No differences at follow-up between the PEL groups were found, except for the cognitive SR skills, where a borderline significant result emerged between low PEL control and intervention group. Within the middle PEL intervention group, there was an increase in cognitive SR skills. Even though the intervention did not achieve its goal and the aims were not attained, further analyses should examine whether changes can be seen in the determinants of children’s EBRBs, especially those of importance for children with low PEL. In addition, a thorough process evaluation may provide insight into the non-significant findings.

## Figures and Tables

**Table 1:** Children’s characteristics by the control and intervention group at baseline (*n* = 802).

		Control		Intervention		*p*-Value
		*n*	Mean ± SD *	*n*	Mean ± SD *	
Child’s Age ^c^		441	5.24 ± 1.06	360	5.14 ± 1.04	0.060 ^a^
		*n*	%	*n*	%	
Child’s gender	girl	203	46.0%	172	47.8%	0.496 ^b^
	boy	238	54.0%	188	52.2%	
Parental educational level ^d^	low	116	29.9%	109	35.4%	<0.001 ^b^
	middle	169	43.6%	143	46.4%	
	high	103	26.5%	56	18.2%	
Municipality	Salo	357	81.0%	306	84.8%	0.040 ^b^
	Riihimäki	84	19.0%	55	15.2%	

* SD, standard deviation; ^a^ comparison using *t*-test; ^b^ comparison using Chi-square test; ^c^ one missing value for age; ^d^ low educational level (comprehensive school, vocational school, or high school), middle (bachelor’s degree or college), high (master’s degree or licentiate/doctor).

**Table 2:** Descriptors for children’s EBRBs and self-regulation skills by control and intervention group.

EBRBs and SR Skills *	Baseline				Follow-Up			
	Control		Intervention		Control		Intervention	
	*n*	Mean ± SD **	*n*	Mean ± SD **	*n*	Mean ± SD **	*n*	Mean ± SD **
Total screen time (min/day)	370	84.87 ± 43.45	303	87.27 ± 44.06	325	88.84 ± 42.47	261	85.37 ± 41.34
Total physical activity ^a^ (min/day)	335	412.68 ± 48.40	282	405.66 ± 48.61	270	418.02 ± 45.34	210	414.42 ± 50.42
Sugary everyday food and beverages (times/week)	307	9.70 ± 6.89	293	10.53 ± 7.84	241	10.21± 8.96	200	9.76 ± 6.88
Sugary treats (times/week)	318	5.86 ± 3.99	299	5.77 ± 3.21	236	7.00 ± 5.34	192	6.99 ± 5.34
Fruit and vegetables (times/week)	323	21.79 ± 10.67	298	22.06 ± 13.12	258	22.26 ± 11.38	200	23.22 ± 13.39
Cognitive SR skills (scale 1–3)	383	2.31 ± 0.39	313	2.27 ± 0.43	324	2.32 ± 0.41	256	2.34 ± 0.43
Emotional SR skills (scale 1–3)	383	2.26 ± 0.51	313	2.25 ± 0.52	324	2.25 ± 0.51	256	2.29 ± 0.53

* EBRBs, energy balance-related behaviors; SR, self-regulation. ** SD, standard deviation.

**Table 3:** Comparison of EBRBs and SR skills between intervention and control, and changes within the groups *.

	General Linear Mixed Model ^c^			Linear Mixed Models with Repeated Measures					
Children’s EBRBs and SR Skills	Comparison between Intervention and Control Group at Follow-Up ^c^			Change between Follow-Up and Baseline in Control Group			Change between Follow-Up and Baseline in Intervention Group		
		(95% C.I.)	*p*-Value	diff F–B	(95% C.I.)	*p*-Value	diff F–B	(95% C.I.)	*p*-Value
Total screen time (min/day) ^a^	−4.20	(−9.86; 1.46)	0.146	4.46	(0.48; 8.44)	0.028	−1.42	(−5.86; 3.01)	0.529
Total physical activity (min/day) ^b^	−0.56	(−6.65; 5.53)	0.858	23.77	(18.57; 28.97)	<0.001	27.30	(21.74; 32.86)	<0.001
Sugary food and beverage (times/week) ^a^	−0.57	(−2.09; 0.96)	0.466	0.51	(−0.42; 1.43)	0.285	−0.79	(−1.77; 0.19)	0.112
Sugary treats (times/week) ^a^	−0.13	(−1.03; 0.78)	0.781	1.20	(0.62; 1.77)	<0.001	1.28	(0.67; 1.90)	<0.001
Fruit and vegetables (times/week) ^a^	1.43	(−0.64; 3.49)	0.176	−0.37	(−1.63; 0.89)	0.565	1.21	(−0.18; 2.61)	0.088
Cognitive SR skills (scale 1–3) ^a^	0.02	(−0.04; 0.08)	0.505	0.01	(−0.03; 0.05)	0.574	0.06	(0.01; 0.11)	0.011
Emotional SR skills (1–3) ^a^	−0.03	(−0.04; 0.10)	0.405	0.004	(−0.04; 0.05)	0.858	0.04	(−0.02; 0.09)	0.195

* (*n* = 645–737, estimates, and their 95% confidence intervals (C.I.); ^a^ models adjusted for gender, age, municipality, and parental educational level; ^b^ models adjusted for gender, age, municipality, parental educational level, and accelerometer wear time; ^c^ models adjusted for gender, age, municipality, parental educational level, (accelerometer wear time in PA as behavior), and baseline value of the outcome.

**Table 4:** Comparison between the intervention and control group by parental educational level and changes within groups *.

		General Mixed Model			Linear Mixed Models with Repeated Measures					
Children’s EBRBs and SR Skills	PEL	Comparison between Intervention and Control Group at Follow-Up ^c^			Comparison between Follow-Up and Baseline in Control Group			Comparison between Follow-Up and Baseline in Intervention Group		
			(95% C.I.)	*p*-Value	diff F-B	(95% C.I.)	*p*-Value	diff F-B	(95% C.I.)	*p*-Value
Total screen time (min/day) ^a^	low	−1.69	(−12.30; 8.92)	0.753	1.95	(−5.74; 9.64)	0.619	−3.42	(−11.23; 4.40)	0.391
	middle	−7.88	(−16.60; 0.84)	0.076	4.05	(−1.87; 9.98)	0.179	−2.00	(−8.57; 4.57)	0.551
	high	−3.73	(−16.13; 8.66)	0.553	7.65	(−0.10; 15.39)	0.053	2.95	(−6.86; 12.76)	0.555
Total physical activity (min/day) ^b^	low	−7.17	(−24.15; 9.80)	0.404	21.41	(11.82; 31.00)	<0.001	22.10	(12.89; 31.32)	<0.001
	middle	1.86	(−11.90; 15.63)	0.787	26.61	(19.56; 33.66)	<0.001	30.89	(22.96; 38.83)	<0.001
	high	−0.77	(−19.96; 18.42)	0.937	21.10	(12.08; 30.13)	<0.001	27.66	(16.37; 38.95)	<0.001
Sugary foods and beverages (times/week) ^a^	low	−0.15	(−2.70; 2.41)	0.909	0.83	(−1.07; 2.74)	0.392	0.10	(−1.71; 1.92)	0.911
	middle	−1.08	(−3.08; 0.93)	0.286	0.61	(−0.75; 1.96)	0.380	−0.88	(−2.26; 0.50)	0.210
	high	−1.34	(−4.14; 1.45)	0.344	0.09	(−1.64; 1.81)	0.920	−1.91	(−4.12; 0.31)	0.092
Sugary treats (times/week) ^a^	low	−0.79	(−2.86; 1.29)	0.454	2.17	(0.97; 3.37)	<0.001	2.22	(1.15; 3.29)	<0.001
	middle	0.52	(−1.19; 2.22)	0.545	0.93	(0.10; 1.75)	0.027	0.74	(−0.17; 1.65)	0.109
	high	−0.07	(−2.32; 2.18)	0.954	0.89	(−0.18; 1.96)	0.103	1.02	(−0.34; 2.38)	0.140
Fruit and vegetables (times/week) ^a^	low	2.99	(−1.00; 6.98)	0.141	−0.14	(−2.75; 2.47)	0.915	1.51	(−0.98; 3.99)	0.235
	middle	0.59	(−2.56; 3.74)	0.710	0.37	(−1.49; 2.23)	0.695	1.43	(−0.61; 3.48)	0.169
	high	1.03	(−3.30; 5.37)	0.638	−1.68	(−3.96; 0.60)	0.149	0.31	(−2.74; 3.36)	0.841
Cognitive SR skills (scale 1–3) ^a^	low	0.11	(0.00; 0.21)	0.051	−0.11	(−0.22; 0.00)	0.052	0.04	(−0.08; 0.15)	0.513
	middle	0.001	(−0.09; 0.09)	0.987	−0.03	(−0.13; 0.06)	0.468	0.10	(0.01; 0.20)	0.038
	high	−0.06	(−0.18; 0.07)	0.380	0.04	(−0.09; 0.18)	0.536	−0.04	(−0.18; 0.09)	0.543
Emotional SR skills (scale 1–3) ^a^	low	0.01	(−0.12; 0.13)	0.921	−0.02	(−0.11; 0.08)	0.750	0.03	(−0.07; 0.12)	0.563
	middle	0.05	(−0.05; 0.15)	0.313	−0.02	(−0.09; 0.05)	0.547	0.04	(−0.04; 0.12)	0.286
	high	0.01	(−0.13; 0.16)	0.861	0.07	(−0.02; 0.16)	0.141	0.03	(−0.09; 0.15)	0.611

* Estimates and their 95% confidence intervals (C.I.); ^a^ models adjusted for gender, age in years, municipality, and parental educational level; ^b^ models adjusted for gender, age in years, municipality, parental educational level, and accelerometer wear time; ^c^ models gender, age in years, municipality, parental educational level, (accelerometer wear time in PA as behavior), and for baseline value of outcome.

## Supplementary Materials

The following are available online at https://www.mdpi.com/2072-6643/12/9/2599/s1, Table S1: Number of children and missing values in each outcome, Table S2: Descriptors for study outcomes by the control and intervention groups and by parental educational level (PEL), Table S3: Adjusted differences and their 95% confidence interval (C.I.) between intervention and control group separated for baseline and follow-up; and adjusted differences between follow-up and baseline for each study group.
