# Supplementary material for: Effects of the Preschool-Based Family-Involving DAGIS Intervention Program on Children’s Energy Balance-Related Behaviors and Self-Regulation Skills: A Clustered Randomized Controlled Trial
Source: Nutrients. 2020 Aug 26;12(9):2599. doi: 10.3390/nu12092599 (PMC7551940; doi:10.3390/nu12092599)
Supplement: Supplementary file 1 [file nutrients-12-02599-s001.pdf]

Supplementary Table S1. Number of children and missing values in each outcome.

Dependent variable (outcome)	Number of children included in mixed models with information at baseline or follow-up, or both	Number of child participants at baseline	Number of child participants at follow-up	Children with missing values for PEL*	Children with missing values for age
Total screen total (min/day)	737	673	586	50	1
Total physical activity (min/day)	680	605	492	40	1
Sugary everyday foods and beverages (times/week)	645	600	441	28	0
Sugary treats (times/week)	654	617	428	28	0
Fruits and vegetables (times/week)	652	612	452	29	0
Cognitive self-regulation skills (scale 1-3)	722	697	580	18	1
Emotional self-regulation skills (scale 1-3)	722	697	580	18	1

*PEL parental educational level

Supplementary Table S2. Descriptors for study outcomes by control and intervention groups and by parental educational level (PEL).

	PEL	Baseline				Follow-up			
		control		intervention		control		intervention	
		n	mean ± SD	n	mean ± SD	n	mean ± SD	n	mean ± SD
Total screen total (min/day)	low	104	94.17 ± 48.01	94	97.82 ± 43.48	81	93.95 ± 41.70	77	98.88 ± 47.94
	middle	147	82.57 ± 41.20	125	83.12 ± 38.56	141	88.42 ± 38.11	113	79.56 ± 35.25
	high	94	76.37 ± 39.17	54	77.52 ± 41.48	80	83.61 ± 45.75	49	79.80 ± 33.87
Total physical activity (min/day)	low	90	412.95 ± 49.96	87	402.09 ± 50.76	62	409.64 ± 48.70	61	402.96 ± 53.42
	middle	139	409.95 ± 50.77	116	409.18 ± 46.54	124	419.91 ± 41.72	93	419.00 ± 49.90
	high	84	416.32 ± 44.41	50	402.06 ± 44.11	71	418.49 ± 48.34	40	420.91 ± 50.12
Sugary everyday foods and beverages (times/week)	low	78	9.97 ± 8.77	88	10.02 ± 7.15	56	10.75 ± 13.01	57	10.95 ± 7.24
	middle	134	9.75 ± 5.97	128	10.27 ± 7.08	108	10.44 ± 8.44	96	9.46 ± 7.31
	high	80	8.93 ± 6.19	53	10.42 ± 6.04	70	9.37 ± 5.36	38	8.84 ± 5.00
Sugary treats (times/week)	low	77	5.77 ± 4.56	92	4.78 ± 3.37	53	8.11 ± 7.62	58	7.09 ± 7.99
	middle	141	5.63 ± 3.62	130	6.35 ± 3.02	108	6.56 ± 4.58	86	7.14 ± 3.57
	high	85	6.34 ± 3.82	53	6.08 ± 2.90	67	7.03 ± 4.37	39	7.21 ± 3.63
Total fruit and vegetables (times/week)	low	80	19.85 ± 10.30	91	21.62 ± 13.00	57	20.18 ± 10.23	61	23.13 ± 12.46
	middle	141	21.10 ± 10.05	129	21.88 ± 13.05	116	22.34 ± 12.20	90	23.32 ± 14.63
	high	87	24.68 ± 11.62	54	25.06 ± 14.26	75	23.73 ± 11.12	41	25.05 ± 12.48
Cognitive self-regulation skills (1-3)	low	113	2.36 ± 0.36	108	2.25 ± 0.43	81	2.29 ± 0.40	82	2.37 ± 0.44
	middle	164	2.26 ± 0.41	143	2.24 ± 0.41	146	2.33 ± 0.41	116	2.34 ± 0.43
	high	100	2.33 ± 0.37	56	2.39 ± 0.45	83	2.32 ± 0.41	49	2.32 ± 0.42
Emotional self-regulation skills (1-3)	low	113	2.25 ± 0.51	108	2.23 ± 0.52	81	2.22 ± 0.46	82	2.27 ± 0.53
	middle	164	2.40 ± 0.52	143	2.52 ± 0.52	146	2.20 ± 0.55	116	2.28 ± 0.52
	high	100	2.31 ± 0.48	56	2.33 ± 0.50	83	2.36 ± 0.47	49	2.38 ± 0.54

Supplementary Table S3. Adjusted differences and their 95% confidence interval (C.I.) between intervention and control group separated for baseline and follow-up; and adjusted differences between follow-up and baseline for each study group

	baseline			follow-up			Control			Intervention				
Outcomes	diff I-C	(95% C.I.)	p-value	diff I-C	(95% C.I.)	p-value	diff F-B	(95% C.I.)	p-value	diff F-B	(95% C.I.)	p-value	p-value interaction	Effect size for interaction
Total screen time (min/day)[a]	3.22	(-4.67 ; 11.12)	0.411	-2.66	(-10.75 ; 5.43)	0.508	4.46	(0.48 ; 8.44)	**0.028**	-1.42	(-5.86 ; 3.01)	0.529	0.053	0.01
Total physical activity (min/day)[b]	-6.45	(-14.83 ; 1.92)	0.126	-2.93	(-11.74 ; 5.88)	0.504	23.77	(18.57 ; 28.97)	**<0.001**	27.30	(21.74 ; 32.86)	**<0.001**	0.307	0.001
Sugary foods and beverages (times/week)[a]	0.82	(-0.75 ; 2.40)	0.293	-0.47	(-2.15 ; 1.21)	0.574	0.51	(-0.42 ; 1.43)	0.285	-0.79	(-1.77 ; 0.19)	0.112	0.435	0.001
Sugary treats (times/week)[a]	-0.05	(-0.96 ; 0.86)	0.910	0.04	(-0.96 ; 1.03)	0.941	1.20	(0.62 ; 1.77)	**<0.001**	1.28	(0.67 ; 1.90)	**<0.001**	0.837	0.001
Fruit and vegetables (times/week)[a]	-0.52	(-2.89 ; 1.84)	0.654	1.06	(-1.45 ; 3.56)	0.398	-0.37	(-1.63 ; 0.89)	0.565	1.21	(-0.18 ; 2.61)	0.088	0.088	0.01
Cognitive SR skills (scale 1-3)[a]	-0.04	(-0.11 ; 0.02)	0.202	0.01	(-0.07 ; 0.08)	0.888	0.01	(-0.03 ; 0.05)	0.574	0.06	(0.01 ; 0.11)	**0.011**	0.123	0.001
Emotional SR skills (scale 1-3)[a]	0.01	(-0.07 ; 0.08)	0.882	-0.03	(-0.11 ; 0.06)	0.530	0.004	(-0.05 ; 0.04)	0.885	-0.04	(-0.09 ; 0.02)	0.195	0.384	0.002

[a] models adjusted for adjusted for gender, age in years, municipality and parental educational level

[b] models adjusted for adjusted for gender, age in years, municipality and parental educational level and accelerometer wear time

diff: estimated adjusted difference

FV: fruit and vegetable
